# Screening of a Protein That Interacts with the Matrix Attachment Region-Binding Protein from *Dunaliella salina*

**DOI:** 10.1155/2013/862450

**Published:** 2013-09-04

**Authors:** Rui Yang, Zhaoxi Li, Yan Lin, Baosheng Yang, Tianyun Wang

**Affiliations:** ^1^Department of Life Science and Technology, Xinxiang Medical University, Xinxiang, Henan 453003, China; ^2^Department of Biochemistry and Molecular Biology, Xinxiang Medical University, Xinxiang, Henan 453003, China

## Abstract

We isolated the matrix attachment region-binding protein (MBP) DMBP-1 from *Dunaliella salina* in our previous studies. MBPs are part of the cis-acting protein family cluster. The regulatory function possibly works through the interaction of the MBPs with each other. In the present study, DMBP-1 was used as the bait in screening the *D. salina* cDNA library for DMBP-1 interactors that could potentially mediate the DMBP-1-regulated functions. A novel MBP, namely, DMBP-2, was identified as a DMBP-1 binding partner. The cDNA of DMBP-1 was 823 bp long and contained a 573 bp open reading frame, which encoded a polypeptide of 191 amino acids. The interaction between DMBP-2 and DMBP-1 was further confirmed through glutathione S-transferase pull-down assays.

## 1. Introduction

Genome project results show that only 5% to 10% of genome DNA sequences are coding regions in the genomic DNA of all eukaryotes. Most of the DNAs are noncoding and confer a large number of important regulatory functions, such as promoter, enhancer, and miRNA [1–3]. Studying the functions of noncoding DNAs and their regulatory mechanisms is necessary. Expression regulation of the gene is performed through the interaction between cis-acting elements and transacting factors. The transacting factor must be isolated and examined to study the regulatory mechanism of a certain cis-acting element.

Matrix attachment region DNAs (MAR) are DNA sequences that bind to nuclear matrices after being digested by restriction enzymes. MARs consist of AT-rich sequences that extend from 200 bp to 2000 bp and contain various AT-rich and structural motifs, such as A-box (AATAAAYAAA), T-box (TTWTWTTWTT), base-unpairing regions, and intrinsically curved portions [4–6]. MARs do not encode any protein and are considered noncoding DNA sequences. MARs have an important role in chromatin folding and influence the adjacent endogenous gene expression as new cis-acting elements in the eukaryotic organism. MARs can also increase the transgene expression level and buffer transgenic silencing when the expression vector is inserted [7–11]. MARs can also function as eukaryotic organism DNA replication origins [12]. In our previous study, we found that the MARs of *Dunaliella salina* can increase the transgene expression level by 1.5-fold to 4.5-fold. The mechanism is not related to the transgenic copy. We also found that the regulatory function of a MAR on a transgene confers a “position effect” [13–15].

The mechanism of the MAR regulatory function is unclear. Experimental evidence that explains this function of MAR is lacking because the transacting factor that binds to MAR has not been discovered. We isolated one MAR-binding protein (MBP), namely, DMBP-1 (GenBank Accession nos. DQ124215, AAZ31075), through *D. salina* *λ*gt11 expression library screening. The ability of the protein located in the nucleus in binding to MAR was demonstrated [16, 17]. 

In the present study, we screened the protein that can interact with DMBP-1 using yeast two-hybrid systems and the GST pull-down technology. We also provided a basis for the study of the MAR regulatory mechanism. 

## 2. Materials and Methods

### 2.1. Algae Strain and Culture

*D. salina *strain UTEX 1644 TEOD was purchased from the University of Texas, USA. The strain was grown as batch cultures in a liquid PKS medium at 26°C under a continuous irradiance of 4500 Lux and with a 12 h light/day condition as described by Wang et al. [13].

The cells of *D. salina *at the logarithmic phase were transferred to a solid medium containing 0.8% agar. The cells were then cultured for 3 weeks until single individual colonies appeared. One single colony was picked and transferred to the liquid medium mentioned above for further culturing.

### 2.2. Plasmid Construction

The DNA (1623 bp) fragment coding for *D. salina* DMBP-1 was amplified from the plasmid pUCm-T/DMBP-1 that was previously constructed in our laboratory. The forward and reverse primers were 5′-CGGCATATGATGGTT CTCGTCTTGTTCGAGAC-3′ with an *Eco* RI site and 5′-CGGG AATTCCTCAGCGGCCTTCTTCTT-3′ with an *Nde I* site, respectively. The PCR product was digested using *EcoR*I and *Nde I.* The product was then inserted into the *EcoR*I and *Nde I *sites of vector pGBKT7 of the yeast two-hybrid system (Clontech), which resulted in an in-frame fusion of *D. salina* DMBP-1. The correct reading frame was confirmed through enzyme digestion and sequencing analysis. The expression vector was named pGBKT7-MBP.

### 2.3. Determination of the Plasmid Self-Activation Activity

The yeast strain AY190 was transformed with pGBKT-MBP through the lithium acetate method based on the instructions on the kit (Clonetech) to check for self-activation. The transformants were diluted at 1 : 10 and 1 : 1000. These transformants were then smeared on an SD/-Trp plate and cultured at 30°C for 72 h until single bacterial colonies appeared. The single colonies were picked and cultured in an SD/-Trp liquid medium overnight. The plasmids were extracted from the yeast and were identified through PCR by using the plasmid as a template.

One single colony (2 mm to 3 mm) was picked and cultured in a 50 mL SD/-Trp/Kan (20 *μ*g/mL) liquid medium at 30°C for 72 h overnight. The optical density was measured at a wavelength of 600 nm. The recombinant yeast strains AH109 and Y187, which contained the pGBKT7-MBP plasmid, were smeared on the SD/-Trp, SD/-Trp/-His, and SD/-Trp/-Ade plates and cultured for 1 week. 

### 2.4. Library Screening

The *D. salina* cDNA library was screened with a bait constructed from pGBKT7-MBP using a large-scale sequential polyethylene glycol/lithium acetate transformation method based on the manufacturer's instructions. 

The yeast cells of the Y190 strain that harbored pGBKT-MBP were transformed with 30 *μ*g of the library cDNA. The cells were plated on a medium that lacked tryptophan, leucine, and histidine (SD/-Leu -Trp -His) but contained 25 mM 3-amino-1,2,4-triazole (3-AT). The cells were then incubated for 4 d to 7 d at 30°C.

The single colonies (>2 mm diameter) were picked from the selective media SD/-Trp/-Leu/-His/-Ade. These single colonies were then transferred to a new SD/-Trp/-Leu/-His/-Ade/X-*α*-Gal medium and cultured at 30°C for 2 d to 4 d in darkness until the diameter of the blue colony was >1 mm. The blue colonies were picked and cultured on selective media SD/-Trp/-Leu/-His/-Ade/X-*α*-Gal again. This procedure was repeated three times to exclude false positive colonies.

A second round of selection on the same medium was performed, and the valid AD plasmids were removed. The colonies that activated both reporter genes in the Y190 strain were further analyzed. The AD-cDNA plasmid that encoded the interaction protein was isolated from the yeast cells using a yeast plasmid isolation kit (Omega, USA). The selected positive AD plasmids were transformed into *Escherichia coli* DH5*α* cells for further DNA sequencing and BLAST analysis.

### 2.5. Cloning of Full-Length DMBP-2

A partial cDNA that encodes a peptide that interacts with the DMBP-1 of *D. salina* was isolated through a yeast two-hybrid screening of the cDNA library and the designated DMBP-2. Rapid amplification of the cDNA ends (RACE) PCR was performed to obtain a full-length copy of DMBP-2 using a SMART RACE cDNA amplification kit (Clontech) according to the manufacturer's instructions. 

The primer was designed and synthesized for 5′-RACE of DMBP-2 based on the sequencing result of the 3′cDNA end. About 5 *μ*g of the total RNA extracted from *D. salina *was reversely transcribed using primer RT (5′-(P) GGCTCGCAGTTGTAGCCGTACTC-3′) at 30°C for 10 min, 50°C for 30 min, and 80°C for 2 min. The hybrid RNA was denatured and circulated based on the kit protocol. Primers A1 and S1 (A1: 5′-ATG CAGATGATGC AGAAGACC-3′; S1: 5′-TTA TGCAAGGGAA GAGCTTGC-3′) were used for amplification using 5 *μ*L circulated cDNA as the template in a 50 *μ*L reaction system under standard PCR conditions. The PCR products were subjected to electrophoresis, gel extraction, ligation, transformation into *E*. *coli*, and sequencing.

### 2.6. GST Pull-Down Assay

The full-length cDNA encoding DMBP-1 and DMBP-2 were amplified through PCR and subcloned into pGEX-4T-1 (Amersham Biosciences, USA), respectively. The recombinant proteins GST-DMBP-1, GST-DMBP-2, and protein GST were individually expressed in an *E. coli *DH5*α* cell. The soluble proteins were purified as previously described [11] and tested through Western blot using an anti-GST monoclonal antibody. GST-DMBP-1 or GST of up to 150 *μ*g was mixed with glutathione agarose for approximately 4 h. A total of 150 *μ*g His-DMBP-2 was then added to glutathione agarose and incubated at 4°C overnight under rotation. The beads were collected through centrifugation, washed thrice with 300 *μ*L binding buffer, resuspended in the SDS/PAGE sample buffer, and boiled for 5 min before loading on a 10% denaturing polyacrylamide gel. The proteins were analyzed through Western blot with mouse monoclonal antibodies against GST and His-tag (Zhongshan, China).

## 3. Results

### 3.1. Construction of Plasmid and Detection of Self-Activation

DMBP-1 gene was amplified through PCR technology using the plasmid as template and the corresponding primers and was identified through enzyme digestion and sequencing. The gene was then cloned into a pGBKT-7 vector to form the “bait” pGBKT-MBP plasmid.

The pGBKT-MBP and pGBKT-7 plasmids were transformed into yeasts Y187 and AH109, respectively. The results showed that these plasmids could only grow on an SD/-Trp/X-*α*-Gal medium and appeared to have a white color. The plasmids could not grow on both SD/-Trp/-His/X-*α*-Gal and SD/-Trp/-Ade/X-*α*-Gal media, which suggests that the recombinant plasmid could not activate the reporter gene His and Ade expression; it also had no self-activation effect. The OD600 of the Y187 transformed strain was greater than 0.8, which indicates that the recombinant plasmid had no toxicity.

### 3.2. Yeast Two-Hybrid Screening Assay

**Figure 1:**
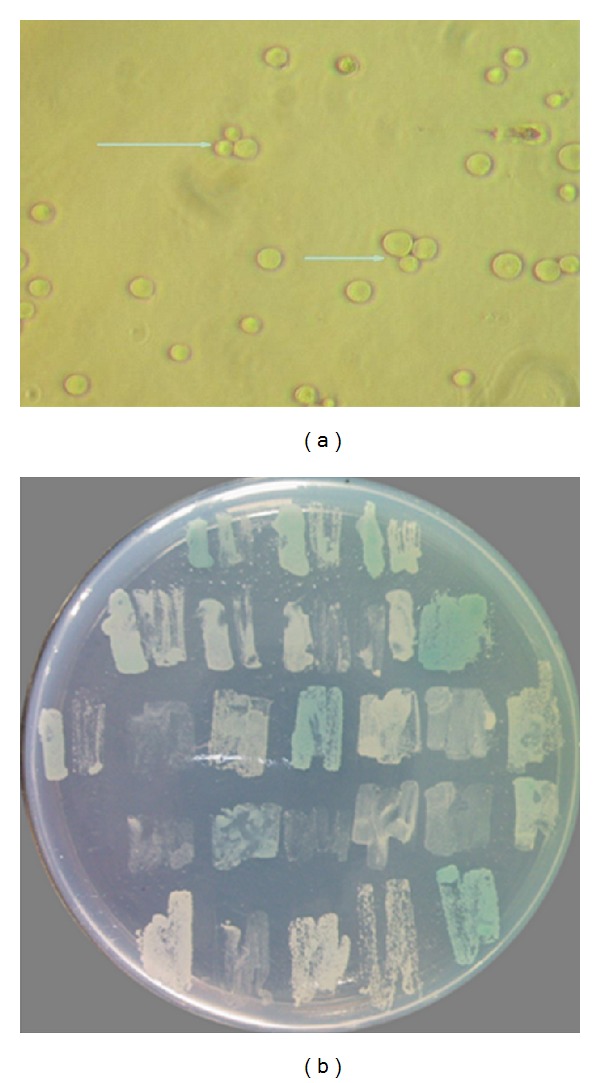
(a) The diploid zygote of the yeast (400x); (b) primary screening of the clone (blue for positive clones).

The “three-leaf grass type” heterozygote was visible under microscope observation 20 h after yeast hybridization (Figure 1(a)). A total of 26 single colonies with diameters of >2 mm were obtained after the primary screening on the SD/-Trp/-Leu/-His/-Ade medium. Seven colonies exhibited blue colors after the second screening on the SD/-Trp/-Leu/-His/-Ade/X-a-Gal medium (Figure 1(b)).

**Figure 2:**
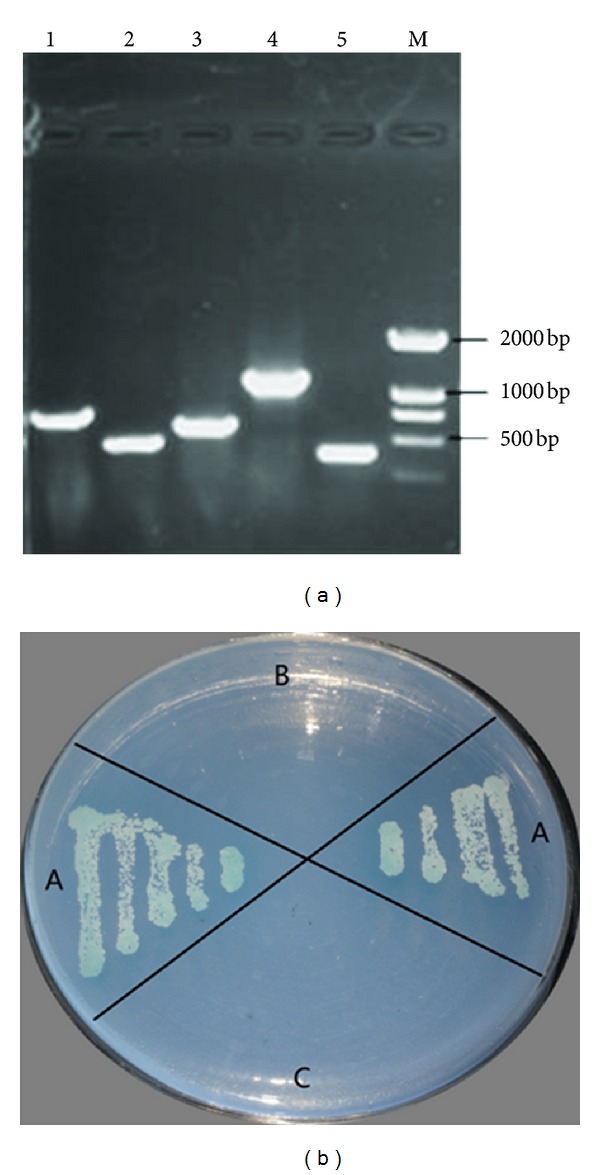
(a) PCR product of positive clones (AD Primer). M: Marker DL2000; 1–5: PCR product. (b) Additional yeast two-hybrid test for the positive clones. A: positive clones; B: negative control (pGBKT-7); C: negative control (pGBKT7-Lam).

 The positive colonies were identified using the library plasmid universal primers (T7 and 3′AD). The length of the inserted DNA fragments was then determined (Figure 2(a)). The library plasmids were transformed into *E. coli* DH5*α*, and the library plasmids and bait plasmids were separated. The positive colonies underwent an additional one-to-one hybridization. The results showed that these colonies retained the blue color on SD/-Trp/-Leu/-His/-Ade/X-*α*-Gal, which suggests that the screened colonies were positive colonies (Figure 2(b)).

The seven positive colonies obtained were sequenced and analyzed through http://www.ncbi.nlm.nih.gov/blast. The results showed that three colonies were homologous with the *Volvox carteri* Fnagariensis photosystem I reaction center subunit VI gene (GenBank no. XM_002956842, 100%), photosystem I P700 chlorophyll a apoprotein A1 ATP synthase CF0 C subunit (GenBank no. GQ250046.1, 98%), and* D. salina* strain UTEX 1644 26S ribosomal RNA gene (GenBank no. DQ015744.1, 100%). The remaining four genes were unknown function genes (GenBank nos. JN006966, JN006968, and JN006967).

### 3.3. Identification of the Open Reading Frames (ORFs) of Selected Genes through RACE

The 5′-upstream sequences of the selected cDNA fragments were determined using RACE technology. Only one gene (PA2) was successfully cloned. The other two were not cloned, possibly because the genes were induced under strong light. The new cloned gene was named DMBP-2 (GenBank no. JN006968).

**Figure 3:**
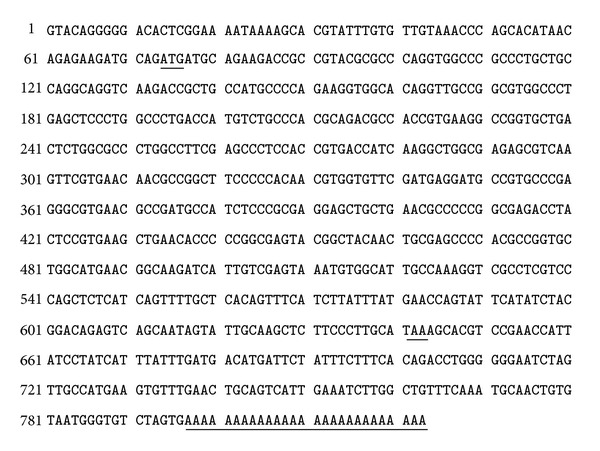
Nucleotide sequence of the gene encoding the DMBP-2 protein from the green unicellular alga *D. salina* and the deduced amino acid sequences (GenBank accession number JN006968). The putative initiation codon (ATG) and the putative polyadenylation signal are underlined. The stop codon (TAA) in italics is underlined. TGAA represents the typical polyA signal sequence.

The 823 bp DNA band was amplified through PCR. The sequencing result of this sequence showed that the cDNA of DMBP-2 possessed a 573 bp ORF from 68 bp to 640 bp of the sequence, which did not include 67 bp 5′UTR and 183 bp 3′UTR. The 3′UTR possessed a typical polyadenylation signal TGTAA at position 45 upstream of the poly (A)-tails (Figure 3).

The DMBP-2 sequence was further confirmed through RT-PCR and sequencing. The results showed that the sequence was correct. We performed the sequence alignments through NCBI Blast (http://www.ebi.ac.uk/). The data revealed that the sequence was highly similar to that of the high light-stressed *D. salina* cDNA with the mRNA sequence EM_EST:CX160991 [18].

### 3.4. GST Pull-Down Assay

**Figure 4:**
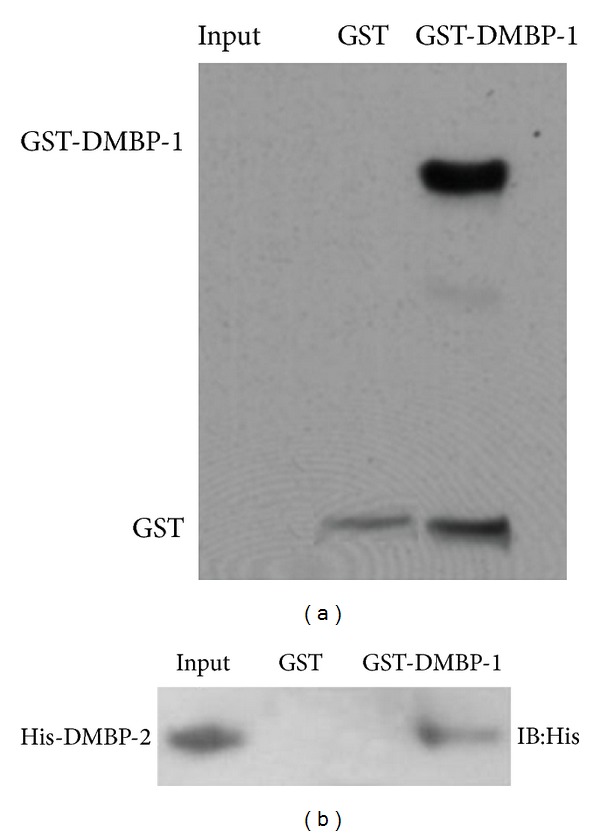
GST pull-down assay demonstrating the in vitro binding between DMBP-1 and DMBP-2. Purified His-DMBP-2 protein was added to GST. GST-DMBP-1 was immobilized on glutathione sepharose beads. His-DMBP-2 fusion protein at 10% was loaded as input. The interacting complexes were subsequently eluted and separated by SDS-PAGE (12%). (a) The immunoblot with monoclonal anti-GST antibody showed the presence of the GST-DMBP-1 fusion protein co-eluting with His-DMBP-2 from the glutathione sepharose beads. (b) The immunoblot with monoclonal anti-His antibody showed the presence of His-DMBP-2 coeluting with GST-DMBP-1 from glutathione sepharose beads. The presence of His-DMBP-2 in the input was also confirmed.

The GST pull-down approach was used to validate the interactions between the newly identified DMBP-2 and DMBP-1. The DMBP-2 protein was mixed with glutathione agarose bound to either a GST protein or to GST-DMBP-1 fusion proteins. The unbound proteins were removed, and the remaining bound proteins were detected. The results showed that GST-DMBP-1 and the GST protein were bound to the glutathione beads (Figure 4(a)). His-DMBP-2 was bound to glutathione beads that contained GST-DMBP-1 (Figure 4(b)), indicating that DMBP-2 directly interacts with DMBP-1.

## 4. Discussion

Some MAR-binding proteins have been isolated from different organisms in the past few years. These proteins are divided into several categories, such as MAR-binding filament-like protein, AT hook-containing MAR-binding protein, and nucleolar protein family. However, these proteins are isolated from higher plants and animals and have been demonstrated to regulate gene expression, influence cell development, induce cell apoptosis, and participate in chromosome assembly [19–22].

Two issues on the study of MAR-binding protein exist. First, the organisms are obtained from higher plants and animals and have tissue differentiations. The gene expression manner and the kinds of tissues are different. The MAR-binding proteins could also be different. Therefore, single-cell eukaryotes may be more suitable in studying the MAR-binding protein. Second, current studies mainly focus on a single isolated MAR-binding protein. Trans-acting proteins, which are protein clusters that contain different proteins, are ignored. Previous research has demonstrated that trans-acting proteins contain more than one protein and interact with each other to fulfill their regulatory functions. For example, the transcription factors include several kinds of proteins. Therefore, we propose the isolation of MBP from single-cell eukaryotes and the further study of the regulatory mechanism of MBP. *D. salina *is a specific single-cell green algae that has no cell wall, contains one big goblet chloroplast that can perform photosynthesis function, has a flagellum, can move and survive at a higher NaCl concentration medium, confers good resistance, and has ideal resistance [13]. The simple and cheap culture of *D. salina* means that it has great potential in bioengineering for producing valuable polypeptides and proteins. In addition, the bias of codon usage of algae is more similar to human than *E. coli* and yeast [23].

Yeast two-hybrid, which was first applied by Fields and Song [24], is a high-sensitivity technology that studies protein interactions; it has been used to identify numerous protein interactions. In this study, we successfully constructed the plasmid pGBKT7-MBP, which contains the *D. salina* DMBP-1 gene. We then transformed the yeast strains AH109 and Y187 and smeared the colonies containing the “bait” plasmid on the SD/-Trp, SD/-Trp/-His, and SD/-Trp/-Ade media. The results showed that the colonies only grew on the SD/-Trp medium, suggesting that the “bait” plasmid cannot activate the reporter gene His and Ade and that it has no self-activation effect. The transformed yeast strain was successfully identified through PCR using the extracted yeast plasmid as template. The single colonies were cultured in an SD/-Trp liquid medium overnight for the toxicity experiment. OD_600_ was greater than 0.8, which suggests that the bait plasmid had no toxicity to the yeast strain.

 Strict screening and hybridization were repeatedly performed. We then obtained six positive library plasmids, which possibly encoded the proteins that could interact with DMBP-1. The screened proteins include the nagariensis photosystem I reaction center subunit VI and the photosystem I P700 chlorophyll a apoprotein A1 ATP synthase CF0 C subunit. The full-length of one gene was cloned through 5′-RACE and possessed 573 bp ORF from 68 bp to 640 bp of the sequence. The sequence alignments show that the sequence is highly similar to that of the high light-stressed *D. salina* cDNA sequence (EM_EST:CX160991) [18].

We performed in vitro binding assays using GST pull-down assays to further confirm the specific and direct interactions between DMBP-1 and DMBP-2. 

In summary, this study screened an MBP that interacts with DMBP-1 by establishing a link between DMBP-1 and DMBP-2. DMBP-1 was found to be a potential candidate in investigating the molecular mechanisms of MBP in regulatory functions.
